# Positivity in the paramedic workplace: pathways to flourishing amidst adversity

**DOI:** 10.1186/s12913-026-15117-6

**Published:** 2026-07-21

**Authors:** Christine Janse van Rensburg, Marais Christel

**Affiliations:** Vaal University of Technology, Human Resource Management, Vanderbijlpark, South Africa

**Keywords:** Flourishing, Emotional well-being, Psychological well-being, Social well-being, Paramedics

## Abstract

**Background:**

Paramedic practice is evolving worldwide. Frequent exposure to traumatic and stressful incidents, combined with demanding workplace conditions, significantly affects the well-being of paramedic personnel. This study aimed to explore the lived experiences of flourishing among private-sector paramedics in post-COVID-19 South Africa.

**Methods:**

The theoretical foundation of this study is rooted in phenomenology. This qualitative approach seeks to explore the lived experiences of individuals willing to share first-person accounts of being paramedics in a post-COVID work context. To address the research problem, a purposive sample of 13 private sector paramedics was selected. Data was gathered through semi-structured interviews using open-ended questions, encouraging rich, reflective accounts of their lived experiences. Thematic analysis in MAXQDA software was used to identify recurring patterns, emergent themes, and unique perspectives.

**Results:**

Workplace flourishing was experienced as a dynamic, interconnected process in the work environment. Findings demonstrated an ongoing interplay between emotional regulation, vocational meaning and relational embeddedness within a high-risk operational context. Flourishing emerged through internal emotional steadiness, professional engagement, and supportive social environments. These findings indicate that flourishing is possible even in high-stress paramedic environments.

**Conclusion:**

Outcomes revealed that promoting well-being among paramedics in the post-COVID period requires integrating emotional support, satisfying psychological needs, meaningful learning opportunities, relational connections and enabling working conditions.

## Background

Paramedic practices are undergoing substantial change internationally, with scopes broadening, community expectations increasing, and emergency care being delivered in new ways [1]. There are serious occupational issues facing paramedic staff on the front line, for example, the severe psychological and physical toll exerted by frequent exposure to traumatic and stressful incidents combined with demanding workplace conditions [2]. South African emergency service personnel regularly face traumatic and life-threatening situations that have a profoundly negative influence on them, yet they are often required to make quick decisions and administer effective aid [3].

The health and well-being of paramedics is not just an individual concern; it can also negatively affect the quality of care that patients receive and the efficiency of health systems [4]. The stressful work environment of frontline health care workers can trigger mental health effects such as depression, anxiety, traumatic stress symptoms, demoralisation, sleep difficulties, lack of functionality and an increase in irritability [4]. These findings highlight the importance of initiatives to support the mental well-being of emergency care staff for the provision of quality care.

Across South Africa, paramedics are sometimes being robbed, assaulted, raped or even murdered [5]. This exposure to workplace violence in hostile environments impedes their ability to flourish within their work context. The notion of flourishing, as a key component of a positive work context, acknowledges the multidimensional nature of employee well-being [6]. Busser et al. [7] state that flourishing relates to subjective well-being, which centres on how people assess their encounters in various workplace settings. Well-being in the work context consists of emotional, psychological, and social dimensions. Emotional well-being comprises, amongst other things, job satisfaction and positive and negative effects [8–10]. Psychological well-being entails autonomy, competence, relatedness, learning, personal growth, meaning, purpose, and engagement [7–9, 11]. Social well-being refers to experiences focused on social tasks and connections encountered in the workplace [11, 12].

Flourishing employees experience a sense of fulfilment from their employment and experience frequent positive effects, including feelings of opportunity and active participation in decision-making when completing assignments, effective management of their condition, and a sense of belonging to associate with others [13, 14]. Finding their work important and deliberate, employees were, at the time, emotionally, intellectually and physically profoundly engaged in their work. When employees flourish, they accept that they are valuable organisational members, representing uplifting perspectives towards their potential [13].

### South African emergency medical services system

South African Emergency Medical Services (EMS) include both the public and private providers. This service is an integral part of the national health system and faces significant structural and operational challenges that affect paramedics’ well-being [15]. Recent research also indicates non-functional components of the EMS system, with issues spanning leadership, resources, provider preparedness, health and safety, training, and effective communication [15, 16].

South African paramedics play a crucial role on the frontline of healthcare, providing pre-hospital emergency medical care to patients outside hospitals [17]. They are highly trained medical professionals who carry out medical assessment, stabilisation, and the safe transfer of sick and injured patients in a range of prehospital environments, such as their homes, workplaces, or public areas, to and from health facilities [15]. Beyond traditional trauma response, paramedics also manage critical care transfers for critically ill patients who require specialised care and ongoing monitoring during interfacility transfers [18].

Paramedics are trained at different levels: Basic Life Support (BLS), Intermediate Life Support (ILS), and Advanced Life Support (ALS) [19]. BLS paramedics provide basic medical care interventions such as cardiopulmonary resuscitation (CPR), stopping of bleeding, assisting women during childbirth and non-invasive procedures. ILS paramedics assist with intermediate medical interventions, including IV therapy or drips, bronchodilators, defibrillation and chest decompressions. ALS paramedics are pre-hospital advanced life support providers with a more complex scope of practice, including advanced resuscitation, advanced airway management, IV drug therapy up to schedule seven drugs, advanced midwifery, and specialisation in aviation and marine medicine [20].

### The paramedic context

#### Transitional experiences

The transition from college to professional practice presents additional hurdles for newly qualified emergency care staff [21]. They reported poor practice in specific workplaces in how they are received, with many feeling excluded and encountering resistance from colleagues. However, some participants reported that peer support, positive feedback and acquiring new skills during training help boost professional confidence once in employment [21].

#### Gender disparities

Makkink and Nkhoma [22] discovered that female paramedics experienced gender discrimination and had more mental health implications than their male colleagues. A climate of gender discrimination presents stressors that are not characteristics of the occupation. Female paramedics reported psychological stress, feelings of inadequacy, isolation, sadness, and questioning themselves in the work context. Females also experienced greater intersectional responsibilities as they navigated gender-based discrimination while juggling broader societal expectations regarding motherhood and family responsibilities [22]. This, in turn, leads to impossible binds where female paramedics are forced to choose between family involvement and career progression, whereas their male counterparts usually don’t face the same trade-offs.

#### Exposure to traumatic events

South African paramedics and EMS personnel face specific working stressors within the broader healthcare system. A study discovered that 30% of ambulance workers in South Africa show signs of post-traumatic stress disorders, with persistent work-related stress being a key risk factor [23]. Another study involving first responders, conducted by Padmanabhanunni and Pretorius [24], found that exposure to traumatic events in the line of duty is an intrinsic risk factor for the development of work-related burnout. The experience of a build-up of exposure to traumatic events (e.g. paediatric deaths and mass casualty incidents) results in both acute and cumulative trauma, which has adverse effects on paramedics’ physical health. Physical occurrences such as headaches, insomnia, musculoskeletal ailments, exhaustion, nutrition issues, weight gain and exposure to hazardous microorganisms were also reported [25].

#### Workplace violence and bullying

Exposure to workplace violence creates additional trauma and safety concerns for paramedics. Violence also hurts access to emergency medical care and further compromises the well-being of paramedic staff [26]. According to research by Makkink and Nkhoma [22], this occupational hazard remains inadequately addressed, and female paramedics particularly report vulnerability to gender-based violence and sexual harassment in workplace environments. The additional threat of violence, aggression and even verbal assault that paramedics face may contribute to their feelings of work-related vulnerability and uncertainty. This is only exacerbated when concerns are not reported or under-reported, as potential aggressiveness and even violence may be perceived as “part of the job” within many cultures [25]. Workplace bullying, physical assault by others such as patients or colleagues, verbal and physical threat from others, lack of backup support and evidence that incidents are not taken seriously or are not investigated are similarly prevalent in regard to feelings of loss of control or safety [27].

### Work-life balance

Ambulance work is inherently unpredictable. In addition to the nature of the work itself, workload, work demands, shift work, limited time for debriefing or downtime, the hierarchical nature of supervision, and a lack of recognition have all been shown to create challenges to work-life balance, with negative consequences for family relationships and personal health [3, 25, 28].

Paramedics depend on their families for emotional support. Paramedicine is a male-centric culture that impedes the expression of emotion in the workplace and places additional burdens on family relationships [28, 29]. When paramedics are consistently absent from family events, unable to maintain a parental presence and too emotionally drained to interact effectively, family relationships become strained. Partners and children receive less of the paramedic’s time, attention and emotional energy, leading to feelings of abandonment or resentment [2, 28].

Other systemic shortcomings, such as fragmentation into districts, inefficient workforce, and suboptimal fleet management, also put paramedics in a position where they struggle with work-life balance [15].

### Social support systems

Social support is a multi-level (family, peer, organisational) phenomenon that mitigates the negative impact of trauma exposure and occupational stress [29]. In a study of South African healthcare workers, practising mindfulness activities and self-care behaviours were significantly associated with well-being, while coping mechanisms for stress and connection to social support networks were significantly associated with both well-being and resilience [30].

Effective peer support networks, such as peer support programmes, mentorship relationships, and informal collegial relationships, all show protective effects to buffer health care workers against occupational stress effects [31]. Communities of practice where employees can process shared occupational experiences, discuss challenging cases, and normalise occupational stress responses reduce psychological distress and enhance resilience.

Leadership within the organisation and the support provided by supervisors play a significant role in reducing healthcare workers’ occupational stress challenges. South African research shows that health care workers who report low supervisor support and poor workplace culture have higher levels of burnout, depression, and anxiety [32, 33]. On the other hand, workplace contexts with leader commitment, open communication, valuing staff contributions and sincere concern for worker well-being have been found to buffer mental health problems, reduce burnout, increase job satisfaction, and lead to lower levels of depression and anxiety.

### Workplace flourishing

Workplace flourishing has become an important area of study in modern-day organisational psychology and can be viewed as a state of optimal functioning [34, 35].

Martin Seligman’s PERMA model [9, 36] changed how flourishing is understood in positive psychology. Instead of construing flourishing as a single dimension, Seligman’s model includes five dimensions that embody a flourishing life. The acronym PERMA represents positive emotion, engagement, relationships, meaning, and accomplishment, interrelated dimensions that encapsulate the multifaceted nature of flourishing.

Positive emotion, which can be seen as an emotional well-being dimension, is characterised by happiness and pleasant affective experiences. Engagement is described as a state of being absorbed and focused on activities that are challenging and use one’s skills, also known as “flow”. Relationships, which can be viewed as a form of social well-being, highlight the value of positive interpersonal connections and meaningful social ties. Meaning is the perception that one’s life has purpose and direction and transcends oneself. Finally, accomplishment refers to the human motivation to accomplish goals, develop competence and gain a sense of efficacy [9, 36, 37]. Engagement, meaning and accomplishment are particularly relevant to psychological well-being. Furthermore, recent studies have shown that the original PERMA model has matured in multiple ways. The framework has been extended to PERMA + 4, which includes physical health, mindset, work environment and economic security as supplementary dimensions specifically applicable in organisational environments [38].

Tigedi et al. [10] found that employees who experience workplace flourishing reported high positive emotions, strong job satisfaction, increased autonomy, competence, and greater meaningful engagement with their work. A systematic review by A’yuninnisa, Carminati and Wilderom [39] revealed that flourishing at work is consistently associated with increased engagement, resilience and organisational citizenship behaviour. A study by Peethambaran and Naim [40] indicates that flourishing enhances development and growth, ultimately supporting accomplishments. Bosomtwe and Nubuor [41] established that flourishing employees are associated with better task performance and lower turnover intentions. Other empirical studies demonstrate that flourishing predicts increased performance and decreased emotional exhaustion, suggesting that organisational climate factors such as support, autonomy and meaningful work can cultivate flourishing [42, 43]. Moreover, flourishing is not just a personal experience but also a shared one, with team-level flourishing adding to organisational sustainability [44]. Muñiz-Velázquez et al. [45] utilised the PERMA profiler and discovered that flourishing or happiness at work emerges when employees build strong relationships with their colleagues. In developing country contexts, such as South Africa, flourishing has been demonstrated to manifest uniquely with workers emphasising relational and communal aspects of well-being [46, 47].

The above-mentioned research indicates that paramedics are affected by various aspects and components of their work context. The study, therefore, aims to raise awareness of paramedics’ first-person accounts of flourishing amid the challenges of their work context.

## Methods

This research used a qualitative phenomenological design to explore the lived experience of a selected group of private-sector paramedics in the post-COVID work context. The aim was to capture the essence of a phenomenon by investigating it through first-person accounts from individuals who have worked as the first line of medical professionals who attend to patients in need of emergency medical services.

### Setting and participants

The demarcated area of the study was an emergency care ambulance service based in southern Gauteng. Purposive snowball sampling was used to enable the researcher to utilise her judgement in selecting an initial 13 private paramedic participants for data collection purposes. An initial contact referred the researcher to more potential participants with first-hand knowledge of the phenomenon under investigation.

### Data collection

A Google document information leaflet link was sent to the company’s head office, and all paramedics were made aware of it. An interview guide was developed for this study by the research team. Available paramedics were contacted to schedule one-on-one and online interviews. Interviews were conducted in a privately agreed-upon setting via Zoom with off-duty paramedics and at the ambulance service offices with on-duty paramedics. Semi-structured interactions were guided by open-ended questions and an adaptable interview protocol, and were enhanced with follow-up questions, probes, and comments. Interviews were audio‑recorded with participants’ permission and were transcribed by a professional transcriber. Transcripts were anonymised and stored on password‑protected Google Drive files, accessible only to the research team.

The final sample included 13 participants, which aligns with qualitative research guidelines suggesting 6–12 knowledgeable participants to explore lived experiences [48]. Recruitment and analysis occurred simultaneously, with continuous coding after each interview. Data saturation was first observed after the 11th interview, at which point no new themes or insights emerged. To confirm saturation, two additional interviews were conducted. Saturation was defined not only by the absence of new codes or themes but also by a lack of new nuances within existing codes. The research team identified saturation during the analytical process through progressive coding, emerging themes, and reflective discussions. Additionally, a coding framework, team debriefing, and reflective field notes contributed to a clear audit trail and enhanced the rigour and trustworthiness of the analysis.

### Data analysis

A naturalistic data transcription approach yielded extensive verbatim reports of the joint meaning-making process. Coding was built on a rich, dense, and non-numeric foundation. The researcher gained a deeper understanding of the participants’ thoughts and viewpoints by reviewing transcriptions. This approach used an open inductive coding process whereby codes emerged as the data “spoke for itself”. Transcripts were uploaded using the qualitative data analysis software, MAXQDA. This programme assisted in identifying recurring patterns, emergent themes, and unique perspectives, and in creating an audit trail to improve the investigation’s trustworthiness.

### Ethical considerations

Formal permission to conduct the research was sought from and granted by the university’s Research Ethics Committee (approval number FRECMS-20082025-229). All participants received written information about the study and provided informed consent prior to participating in the interviews. Confidentiality was preserved by anonymising transcripts and quotations, and pseudonyms or participant numbers were used in reporting. Ethical practice throughout the study adhered to established norms for qualitative social research, including respect for participants’ autonomy, sensitivity to potential emotional distress when discussing work pressures, and clarity about voluntary participation and withdrawal. Furthermore, the researchers adhered to a neutral approach and avoided exerting their personal characteristics and beliefs on the results of the study [49]. The researchers, as methodologists-in-action [50], designed, organised, conducted, and presented the study findings with scholarly openness, interpreting the participants’ account of meaning-making in their daily lives in a post-COVID workplace reality.

## Results

**Fig. 1:**
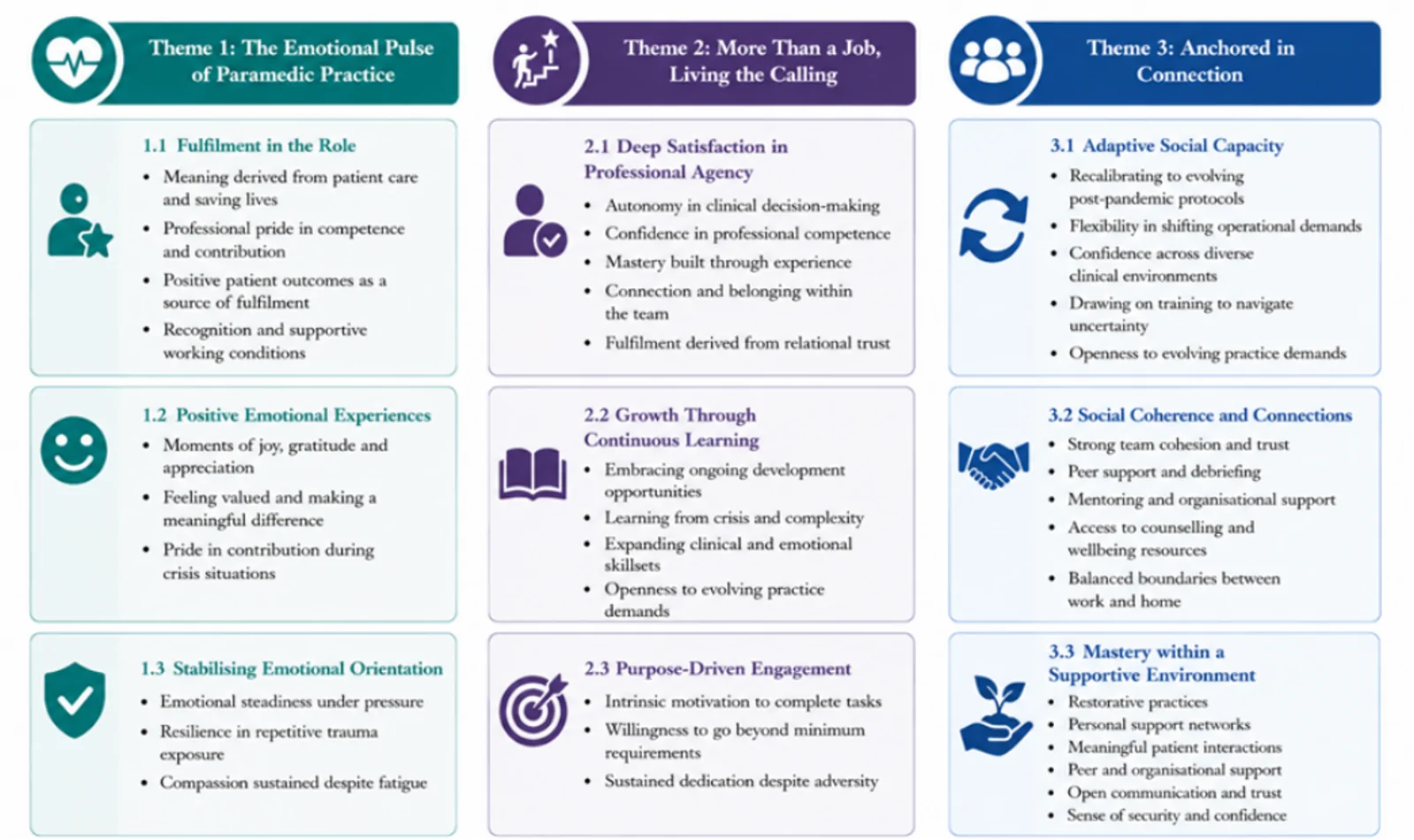
Representation of unfolding findings. Note: Image generated using CHATGPT (Version 5.0), from the author-supplied prompts, 2026

Workplace flourishing was revealed as a dynamic, connected process, rather than isolated experiences in the work environment. Participants’ accounts demonstrated the ongoing interplay between emotional regulation, vocational meaning and relational embeddedness within a high-risk operational context. Flourishing emerged as the convergence of internal emotional steadiness, professional engagement, and supportive social environments, presented as three interrelated domains (Fig. 1). Each sub-theme is further elaborated through categories that capture core dimensions of experience informed by participant narratives.

### Sub-theme 1: The emotional pulse of paramedic practice

Emotional well-being in the post-COVID paramedic context emerged through participants’ experiences of professional fulfilment, positive emotional engagement, and adaptive emotional regulation. Participants’ accounts revealed that flourishing was not characterised by the absence of stress, but by their capacity to sustain growth, connection, reconnection and emotional regulation within demanding operational circumstances.

### Fulfilment in the role

Participants associated emotional flourishing with a sustained sense of personal growth, competence and relational support within their work contexts. Emotional fulfilment was closely linked to opportunities for ongoing learning, expanded professional responsibility and a sense of being recognised. Continuous professional development emerged as a key contributor to emotional well-being. Participants described structured learning environments characterised by ongoing training, updated protocols and opportunities to strengthen clinical competence. Participant 10 explained: *“Continuous training that doesn’t stop when you’ve obtained your qualification … they supply us with constant knowledge and updates. Make sure we are up to standard.”*

Professional growth also functioned as a source of emotional reward. Opportunities for career advancement and expanded scopes of practice strengthened participants’ sense of competence and professional identity [51]. Participants described increased satisfaction through acquiring higher qualifications, advanced skills, and broader clinical responsibilities. Participant 6 expressed: *“Every time you qualified higher*,* you were able to do more skills … more likely to help.”* Access to supportive learning environments and development opportunities further strengthened participants’ sense of growth and flourishing [52].

Specialisation and post-pandemic training initiatives additionally reinforced participants’ sense of organisational recognition and value. Acquiring specialised skills enabled paramedics to navigate complex situations with greater confidence, thereby strengthening their sense of professional purpose [53]. Participant 11 explained: *“After this pandemic*,* they actually forced us to attend courses … that’s where I see okay*,* ja*,* I’m being valued here. Study*,* study*,* study.”* Collaborative learning opportunities also fostered shared problem-solving and mutual support among colleagues [54].

Beyond professional development, emotionally supportive working relationships contribute to participants’ workplace fulfilment. Participants repeatedly described their teams as close-knit and family-like, particularly in the aftermath of the pandemic. These relationships foster trust, psychological safety, and emotional stability within high-pressure operational contexts [55]. Participant 3 shared: *“Being part of a team… [it] be like a family … if you have a good relationship with your partners … you have a peaceful workplace.”*

### Positive emotional experiences

Participants’ definition of emotional flourishing was rooted in the continuity of positive emotional engagement amid the demands of emergency care work. Their accounts did not just capture the feelings of joy and pride, but a general attitude of optimism, career satisfaction, appreciation, and emotional stability in challenging situations. Recognition and positive feedback emerged as emotional reinforcers [56]. Participants described how acknowledgement from patients, colleagues and supervisors validated their professional contribution and strengthened their morale. Feeling that their efforts had made a meaningful difference enabled participants to maintain a positive outlook despite emotional challenges associated with paramedic practice. Participants acknowledged experiencing joy, pride, gratitude and a sense of value through their work engagement. Joy was especially associated with patient outcomes and life-saving interventions [57]. Participant 3 described: *“Saving lives is what gives me joy … just to see someone living and … get to see another day of their life”*.

Participants further described fulfilment through witnessing patient recovery and knowing that their interventions had improved someone’s condition. These experiences reinforce a sense of pride, purpose and professional value. Participant 7 explained: *“When you’ve made a difference in this person’s life at their worst moment … and they say thank you very much for saving my life. … it does happen.”* Positive feedback validated participants’ skills and professional competence, strengthening their commitment to continued growth and service [58]. Participant 8 similarly noted: *“Knowing that we’re actually making a difference … it adds value.”*

Importantly, participants’ positive emotional experiences did not reflect the absence of trauma or stress. Rather, emotional flourishing appeared to involve maintaining composure, professional objectivity and emotional stability within highly distressing circumstances. Participants described remaining calm in traumatic situations while continuing to function effectively. Participant 6 stated: *“I never had to shy away from blood or people in pieces … It’s part of the job.”* Emotional steadiness was further reinforced through reflection on successful patient outcomes during and after the pandemic. Participant 1 shared: *“The best part … is to see that you have made a difference in somebody else’s life… I’ve had patients where the lady was on a ventilator with COVID*,* and she recovered.”* Collectively, these accounts suggest that positive emotional experiences were sustained through meaningful patient effect, professional affirmation, and the ability to maintain emotional composure in high-pressure clinical environments.

### Stabilising emotional orientation

Paramedics described their emotional mindset as a form of regulated engagement that enabled them to function effectively in persistently high-pressure and often traumatic environments. Their accounts were not linked to emotional detachments, but rather to the ability to modulate emotional responses while maintaining clinical clarity and professional composure [59].

A key feature of this emotional regulation was the capacity to remain stable under acute stress, allowing them to think and act appropriately. By effectively managing stress, paramedics safeguard their mental health and thereby enhance their flourishing. Participant 6 revealed: *“I never had to shy away from blood or people in pieces … it’s part of the job.”* This form of emotional stability functioned as a protective mechanism, reducing the risk of emotional overload and supporting sustained engagement in demanding work contexts [60].

Closely linked to this was the practice of emotional boundaries management [52, 61]. Participants described deliberately separating professional experiences from their personal lives in order to preserve their psychological well-being, long-term emotional equilibrium and prevent cumulative fatigue. Participant 12 shared: *“I keep my work and my private life completely separate.”*

Despite this emotional regulation and boundaries, participants did not describe emotional detachment. Instead, they highlighted a form of controlled emotional involvement characterised by empathy and situational sensitivity. Emotional intensity was particularly pronounced in cases involving children or patients resembling their own family members. Participant 6 noted: *“This kid is the same as my kid. It could have been my kid.”* These experiences created emotional resonance, yet participants emphasised the importance of maintaining functional composure in the moment.

Resilience emerged as the mechanism through which emotional demands were sustained over time. Participants described resilience as both a learned skill and a practical coping resource that enabled them to manage ongoing exposure to distressing incidents [60]. Participant 8 explained that coping relied on *“having the tools and having the knowledge of what to do during stressful environments.”* Even in the context of post-pandemic uncertainty and evolving protocols [62], participants described maintaining functional stability through experience, teamwork, and situational adaptation. Participant 9 added: *“We still came back for more*,* because we knew somebody had to do it.”*

Organisational and peer support systems further reinforced emotional sustainability [63]. Access to counselling services and trauma support mechanisms provided additional containment when emotional strain becomes pronounced. Participant 1 divulged: *“If you need a counsellor*,* there will be a counsellor made available for you … and make sure that you’re well and functional … Emotionally*,* there was support.”* Resilience also emerged as a critical tool that helped paramedics to face everyday challenges, to recover from adversity, and safeguard their individual well-being. While encountering new protocols, changing situations, and altered healthcare practices in a post-COVID work environment [62], paramedics managed potentially traumatic situations with composed self-control [60]. Participant 4 described that coping often meant placing emotions *“in the back of my mind”* to continue functioning compassionately and adaptively.

Compassion remained a defining feature of paramedics’ professional identity [64]. Despite experiencing fear or anxiety when arriving at emotionally charged scenes, paramedics continued to engage patients and colleagues with empathy. Participant 10 acknowledged the emotional difficulty of paediatric cases: *“Especially when it comes to children … that’s the one that will make you a little bit emotional.”* Parenthood intensified this response. Such moments triggered deep emotional resonance, yet paramedics regulated these responses to remain clinically effective. Compassion was therefore not a vulnerability, but a disciplined strength that enhanced patient care and collegial support. Participants were not immune to the emotional impact of their work.

Reflective insight emerged through reflective coping strategies. The unpredictable and high-pressure nature of emergency work required composure and rapid decision-making. Humour served as a shared way to cope with extreme situations without becoming overwhelmed. Participant 5 elaborated: *“We will make jokes so that we can forget what you just saw.”* This lightness did not trivialise trauma; instead, it created emotional distance that allowed them to continue functioning. Collectively, hopefulness, compassion, and reflective coping illustrate an emotional mindset characterised by regulated empathy, supported resilience, and sustained psychological balance. The meaningfulness of the context is heightened when paramedics approach their work with empathy and compassion. Understanding the emotions and concerns of patients and their families created a supportive, human-centred atmosphere that fostered social cohesion within the paramedic team.

### Sub-theme 2: More than just a job – living the calling

#### Deep satisfaction in professional agency

Participants described psychological well-being as emerging from a strong sense of agency within their professional roles. In such instances, work was experienced as a deliberate and meaningful life choice rather than an externally imposed obligation. This sense of agency was closely tied to the ability to make autonomous clinical decisions in complex and unpredictable situations, reinforcing both professional confidence and personal commitment [65]. The decision to become and remain a paramedic was again framed as a deliberate life choice [66]. Helping patients was repeatedly positioned as intrinsically rewarding rather than financially motivated. Moreover, the gradual scope of qualification expansion increased this sense of autonomy and professional agency. Participant 6 explained: *“Helping people … every time you qualify higher*,* the more you can do for a patient”*. This progression reinforced a growing sense of ownership, purpose and professional direction.

Autonomy is linked to competence satisfaction, where using skills boosts a sense of mastery. Continuous upskilling leads to better interventions and strengthens both individual and collective mastery. Paramedics feel valued and appreciated in their work, which enhances their sense of relatedness satisfaction. Recognition from the various sectoral stakeholders reinforced participants’ sense of connection and belonging. While expressions of gratitude were described as infrequent, they were nonetheless deeply impactful. This added to a sense of emotional fulfilment despite the demanding nature of the participants’ profession.

### Growth through continuous learning

While certain healthcare protocols and practices have resulted from the COVID-19 environment [67], participants described continuous learning as an essential feature of their professional lives. Rather than being experienced as formal compliance, ongoing development was framed as a practical necessity for remaining effective and responsive in unpredictable clinical environments [51, 68]. In this sense, learning functioned simultaneously as an adaptive strategy and a pathway to flourishing. In addition, ongoing professional development was presented as a professional commitment rather than an obligation.

A central feature of participants’ accounts was the evolving need to integrate technical competence with ethical and relational awareness. Participant 8 described a structured learning intervention that explicitly linked clinical decision-making with empathetic practice: *“The idea of this course is to interlink those … so that people can actually be empathetic towards the patient and still do the right thing.”* Training initiatives were not only seen as skill enhancement but also as shaping how paramedics engaged with patients in complex situations. Participants described how increased exposure to training, advanced procedures, and complex cases strengthened their ability to respond effectively in critical situations [69].

Over time, increased competence fostered trust in participants’ abilities, leading to a sense of accomplishment and fulfilment in their work. Participants expressed that an excellent professional reputation not only gives them pride but also improves their career opportunities, job satisfaction, and overall well-being. Participant 6 explained: *“We are not ambulance drivers as some people think we are.”*

### Purpose-driven engagement

Participants described their work as being anchored in a strong sense of meaning and purpose that extended beyond employment. This reflected a deeply internalised commitment to service and care among the participants. The purpose largely influences participants’ understanding of professional identity and responsibilities, thereby consistently framing paramedicine as a calling rooted in moral commitment and in service to others [52, 70]. This strong sense of purpose meant that paramedics were able to bounce back, remain resilient and still deliver exceptional care to patients [70, 71]. Participant 1 reflected: *“To be able to see that you’ve made a difference in somebody else’s life … is my reward.”* Such experiences reinforced a sense of existential meaning derived from the ability to assist others in critical situations. Participant 9 expressed: *“We opened ourselves to being infected and dying … because we knew somebody had to do it.”* A sense of purpose was therefore further intensified through exposure to risk and uncertainty, especially during the pandemic [72].

The post-pandemic required ongoing adjustment to changing protocols and resource pressure, with participants describing the need to remain focused, responsive, and future-oriented [73]. Participant 4 explained: “*Your challenges are to adapt and just carry on … you can’t sit back and just wait for stuff to happen. You need to adapt to change.”* Statements like these show an internalised moral standard rooted in responsibility and integrity. Understanding their purpose helps paramedics align their decisions with their core values, fostering authenticity. When they live in accordance with these values, they feel fulfilled and thrive [74]. Paramedics are usually the first responders in medical emergencies, providing critical care that can save lives [75]. Participants found their work meaningful as it positively impacts others, adding fulfilment and satisfaction to their roles. Staying committed to their goals required self-motivation.

In addition, paramedics frequently go above and beyond what is required to assist patients [76], and this dedication helps them to advance in their careers and gives them a sense of accomplishment and purpose in their work. Paramedics found great job satisfaction in positively impacting lives by providing patient-centred care, listening to concerns, and involving patients in treatment decisions. They enhanced patient satisfaction and healthcare experiences by integrating feedback into their approach [77]. This promoted collaboration and improved communication among team members, resulting in more efficient and coordinated patient care [78].

### Sub-theme 3: Anchored in connection

Participants’ narratives indicated that social well-being in the post-COVID period was shaped by their capacity to adapt to changing environments, sustain functional relationships and maintain confidence within the organisational systems. Social well-being was therefore experienced not only as interpersonal connection, but as the relationship and structural conditions that enabled effective functioning in complex and evolving healthcare contexts.

### Adaptive social capacity

Participants described social adaptation as a necessary professional competence in response to shifting post-pandemic operational conditions. As healthcare systems transitioned away from strict COVID-19 protocols, paramedics navigated a gradual return to stability, albeit not a full return to pre-pandemic normality. Participant 1 explained: “*We’re very close to [normal]*,* it in the sense of all the precautions that was put in place … it’s much lesser and much more relaxed.”*

Adaptation was further required to address ongoing procedural and environmental variability. This included changes to protective protocols, clinical workflows, and emergency response expectations. Rather than viewing these changes as threats or destabilising, participants embraced them as part of their professional growth, enabling them to operate effectively amid chaos. This adaptive capacity also extended to working across diverse and unpredictable environments. Paramedics reported frequent exposure to varied clinical and geographical contexts, requiring flexibility and situational responsiveness. Participant 7 noted: *“… I’ve been trained to work through things differently than other people*,*”* while Participant 8 added: *“I’m coping very well … having the tools and having the knowledge of what to do during stressful environments.”*

### Social coherence and connections

Beyond individual adaptation, social well-being was strongly shaped by relational cohesion and organisational support structures. Collegial support was frequently framed in familial terms [55]. Team cohesion was frequently associated with trust and co-dependence [79]. Participants described working relationships that enabled coordinated action and mutual reliance in clinical settings. Participant 5 explained: *“We work quite well together*,* we look after each other … that’s really your family.”* Such relational trust supported effective task allocation and strengthened operational reliability in emergency contexts.

Leadership emerged as a crucial component of social well-being. Supportive managerial practices contributed to perceived stability, morale, and workplace flourishing. Participant 13 shared: *“The manager … he’s there to listen*,* and he does sort everything out.”* Similarly, Participant 3 noted the importance of *“having a good manager in your workplace.”* Manager support, as voiced by participants, contributed to a sense of organisational containment and professional support.

Social well-being and work-life balance were shaped by participants’ conscious efforts to maintain functional boundaries between professional and personal domains. While such boundaries were generally described as protective, they were also occasionally experienced as strained under emotionally complex circumstances. Participant 6 illustrated this tension, describing the challenge of maintaining professional functioning while experiencing a personal crisis during a call-out. Such accounts demonstrate the emotional demands embedded within maintaining occupational roles in demanding contexts.

#### Mastery within a supportive environment

Restorative practices and personal support networks helped participants maintain balance amid demanding work conditions. Outdoor activities, hobbies and family support created emotional distance from workplace stress and strengthened coping capacity during and after the pandemic. Participant 6 illustrated how restorative practices created psychological recovery: *“I sometimes go fishing … or do my own garden … your mind goes away from what you are doing here.”* Tjin et al. [80] state that support from friends and family is crucial during difficult times. Participant 12 reflected: *“Yeah*,* family. I get a lot of support … they supported me through COVID.”* These networks reduced anxiety and enhanced coping capacity while facing the challenges brought about by post-pandemic changes in work hours or financial uncertainties.

Social affirmation extended beyond personal relationships and included interactions with patients and broader support systems. Participants described empathy, reassurance and respectful communication as central to meaningful patient engagement. Participant 1 remarked on the emotional significance of witnessing recovery: *“To be able to see people smile again …”*. Such experiences strengthened participants’ sense of contribution and reinforced the relational meaning embedded within paramedic practice.

Formal and informal peer-support systems also contributed to environmental mastery and social connectedness. Access to mentoring, organisational support and broader professional networks strengthens participants’ confidence that assistance was available when required [81]. Participant 6 explained: *“The support from the company … It’s always there. I … Your crews always got your back.”*

Participants further associated flourishing with open communication and constructive team dynamics. Peer support and debriefing following challenging or traumatic occasions provided a sense of belonging and sustained morale [79]. Importantly, communication was described as enabling closeness without eroding professional or personal boundaries. Participant 6 shared: *“I know I can communicate with them … I’ll talk to some about my personal stuff*,* but I don’t have some of them as house friends.”*

Mutual trust, humour, encouragement, and shared resilience further contributed to an uplifting team culture. Participant 7 reflected: *“We try and keep each other positive … you’ll think we’re a bunch of crazy people*,*”* while Participant 5 summarised this collective ethos simply: *“We look after each other.”* Feeling supported by both colleagues and employer structures reinforces security and confidence [82]. As Participant 10 noted: *“You know you have the support from your employer … there’s the procedure in place.”*

Collectively, these accounts demonstrate that mastery within the paramedic work environment was not achieved through independence alone, but through embedded systems of support, communication and relational trust. Social flourishing, therefore, emerged through the interaction between individual coping practices and supportive organisational and interpersonal environments.

## Discussion

This study explored how private-sector paramedics experience workplace flourishing in the post-COVID world. Flourishing at work is not simply the absence of difficulties; instead, it is an ongoing process amid uncertainty and emotional stress related to one’s work. It involves emotional regulation and finding meaning and connection with others, emphasising various aspects of well-being [10, 32]. The results explain flourishing in terms of interconnected aspects such as positive emotion, engagement, positive relationships, meaning, accomplishment, positive mindset, and positive environment. Participants’ experiences indicated that these dimensions did not exist in isolation in their post-pandemic paramedic practice.

### Flourishing in a state of regulation when faced with difficulties

Admittedly, emotional wellness is complex and affects everyone differently. However, emotional flourishing was found to be based on regulated emotional engagement rather than emotional distancing. Paramedics exhibited professional composure during traumatic cases, while preserving compassion, using resources such as peer debriefing, organisational counselling and humour to process trauma without jeopardising care. This aligns with existing literature, which suggests that emotional regulation assists with stress management among emergency healthcare workers [83]. Collective resilience processes in the face of shared trauma and team-based emotional containment served to attenuate the effects of cumulative psychological burden [84]. These results provide evidence that emotional expression, as enabled by organisational and peer systems, supports rather than detracts from professional work. Paramedics face challenges related to paediatric trauma, fears about loved ones, and the pressures of professionalism during personal crises. Consequently, vulnerability and resilience coexist; one does not replace the other. This dynamic is particularly significant in emergency services, where emotional expression may be suppressed by a culture of stoicism. The findings challenge idealised notions of resilience, emphasising that flourishing involves actively engaging with emotional challenges.

### Vocational meaning and professional agency as protective mechanisms

In all narratives, paramedicine was described as a calling rather than just a job, emphasising service, competence and moral duty to preserve life. Professional growth opportunities, broadened scopes of practice, and post-pandemic training enhanced competence, identity, and intrinsic motivation, aligning with the self-determination theory [10]. Research shows that having a purpose at work enhances commitment and job satisfaction [52]. Helping others, preserving life, and acting ethically during the pandemic reinforced participants’ professional identity and engagement. The findings support the self-determination theory concepts of autonomy, competence, and relatedness. Participants experienced growth in agency and mastery through professional judgment, expanded practice, and ongoing training. Learning went beyond organisational compliance; it was a meaningful experience that boosted confidence and identity [85].

Intrinsic motivation-maintained engagement despite post-COVID uncertainties. Occupational strain is also considered a protective factor in international paramedic studies on professional identity and meaningful work [70]. This study highlights how meaning has become more important in the post-COVID private-sector EMS environment, which has undergone procedural changes and increased learning demands. Access to training and specialist pathways within organisations is linked to employee flourishing. While public EMS settings may offer more resources and face different staffing pressures than private EMS settings, a direct comparison is not the focus of this study. Nonetheless, organisational structures appear to significantly influence flourishing and illustrate the need to mediate purpose and fragility as voiced.

### Relational and organisational foundations of flourishing

Flourishing was based on close, trusting relationships between team members and was characterised by shared accountability and co-worker support rather than individual efforts. Team cohesion was both an emotional enabler and operational facilitator, enhancing communication, confidence and integrated care [86]. In the post-pandemic context, teams normalised procedural changes as a form of professional maturation to develop adaptive social capacity and shared meaning-making. Managerial support strengthened psychological safety, whilst networks of support, including families and patients, bolstered purpose and social affirmation [87, 88]. These findings align with existing literature, highlighting that teamwork, peer support, and relational trust are vital for the sustainability and well-being of paramedics [55, 79, 86].

Finally, results also confirm the focus on relationships and environment [10], stressed by PERMA + 4. This is enhanced by having people available to talk to, counselling systems and trust in organisational processes. Organisational systems were thus used as social infrastructure to support psychological safety. In line with this, international EMS research links leadership responsiveness and psychologically safe workplaces with decreased distress and improved workforce retention [87, 89, 90].

The findings support these observations and highlight how post-pandemic EMS processes functioned in South Africa. However, relational flourishing was often accompanied by tension, as some individuals avoided sharing emotions with their families or concealed their feelings to protect them. As a result, connectedness became a selective and strategic process, with therapy and work emphasising the need to navigate emotional closeness while also prioritising self-protection.

### Recommendations, limitations and future considerations

Leaders may find value in establishing environments that acknowledge emotional experiences as part of professional practice. Results indicated that individual coping capacity is insufficient for the flourishing of private sector paramedics. The need for reflective debriefing, the availability of trauma-informed counselling, and visible peer-support networks became apparent in pursuit of long-term well-being. Organisational care and collegial support can aid stress reduction for paramedics and maintain their professional commitment and compassion.

Vocational meaning and opportunities for professional growth also emerged as being strongly associated with workplace flourishing. Continued investment in training, refresher courses, career specialisation pathways and mentorship programs could help strengthen professional identity and facilitate adaptability in future health care contexts. Coupling technical proficiency with moral and empathetic practice might add to paramedics’ sense of mastery and purpose. Recognising professional contributions, both formal and informal, may also help reinforce motivation, resilience, and professional pride.

Supportive working conditions can potently nurture flourishing. Attention to safety protocols, access to resources and equipment, manageable workloads, and opportunities for recuperation can reduce emotional and cognitive strain. Together, these relational, developmental, and organisational supports can create enabling environments where paramedics are more likely to feel valued, connected, and able to flourish amid uncertainty or stressful high-risk healthcare situations.

This study has several limitations that should be considered when interpreting the findings. Data were drawn from paramedics employed by a single private EMS in southern Gauteng, and thus represent a context-specific perspective on flourishing in the workplace. While qualitative enquiry does not purport to provide statistical generalisation, it also acknowledges that the results cannot represent the experiences of all paramedic services in South Africa. Instead, context-specific insight allows readers to assess transferability to other EMS settings, depending on similarities in the organisation, professional or geographical contexts. The sample was also fairly similar, further reducing the diversity of perspectives represented.

Additional constraints were imposed during data collection in active operational environments. Some workplace disruptions, time pressure and participant fatigue were observed during the interviews and may have reduced the depth of some narratives. The virtual interviews were also affected by connectivity quality and the inability to observe non-verbal communication, which may have led to less expressive engagements than in face-to-face interviews. In addition, the cross-sectional design captured participants’ experiences as specific post-COVID moments, thereby limiting the ability to capture fluctuations in flourishing and coping over the period.

The researchers had no prior connections with the EMS organisation or the paramedic profession before collecting data. Despite attempts to remain reflexive and transparent, qualitative interpretations are still shaped by the researchers’ perspectives. This study focused on flourishing, which may have introduced social desirability bias, leading participants to downplay their feelings of distress, vulnerability, or negative experiences. This is particularly significant given the predominantly male sample and should be considered when discussing coping and well-being.

Future research could broaden sampling across EMS services and provinces, increase demographic diversity in sample composition and prioritise in-person data collection. Longitudinal designs can be useful in tracking the development and change in flourishing, coping, and professional identity as workplaces continue to stabilise after the COVID pandemic. A further enhancement of interpretive credibility might be achieved through methodological triangulation, such as observations, reflective journals, or mixed-methods approaches. At the same time, the inclusion of additional stakeholders, such as managers, dispatchers, and support personnel, may also provide a more systemic perspective on the organisation’s approach to paramedic well-being and flourishing.

## Conclusion

This study demonstrates that paramedic flourishing in the post-COVID context is a multidimensionally, relationally situated process, rather than simply a product of successful coping. Flourishing was developed through emotional regulation, professional agency and supportive social setting. This, in turn, highlights the interconnection between emotional, psychological and social well-being. Participants spoke of flourishing in terms of their professional development, intrinsic motivation, rewarding interactions with patients and relational networks, which fostered personal well-being and professional self-identity.

Being able to develop competence under pressure, have autonomy in professional decisions, and have a deeply felt sense of purpose were vitally important for psychological well-being. Patient recognition, care companions’ and organisational leaders’ recognition contributed to commitment and reinforced moral investment in caregiving. Moreover, social and organisational factors were also closely linked to flourishing. Participants expressed confidence in their ability to remain calm, together and caring when working in high-risk, uncertain environments, through trust-based team working, adaptive capacity, supportive leadership and confidence in organisation systems. This study builds on the existing body of research on well-being in emergency health care settings by focusing on the interrelationships between emotional safety, meaningful work and organisational responsiveness. Overall, the practice and culture of the workplace, and the leadership behaviours that support paramedic flourishing, must focus on psychosocial safety, place a high value on continuous learning and human connection, and ensure these are at the heart of professional excellence and resilient health systems, despite post-pandemic changes.

## Data Availability

Direct quotes were used from the qualitative research study. The datasets used and/or analysed during the current study are available from the corresponding author on reasonable request.

## Abbreviations

EMS: Emergency Medical Services
BLS: Basic Life Support
ILS: Intermediate Life Support
ALS: Advanced Life Support
